# Neutral endopeptidase inhibitors SOL-1 and candoxatril counteract kidney fibrosis by reducing myofibroblast formation in mouse UUO model

**DOI:** 10.1186/2050-6511-14-S1-P1

**Published:** 2013-08-29

**Authors:** Marina Aleksinskaya, Jacques Duijs, Jennifer Veth, Bettina Husen, Dania Reiche, Daniel Jasserand, Jo De Mey, Ton Rabelink, Anton Jan van Zonneveld

**Affiliations:** 1 Department of Nephrology and Einthoven Laboratory for Experimental Vascular Medicine, Leiden University Medical Center, Leiden, The Netherlands; 2 Department of ABPH-REL/CCS, Established Products, Abbott Products GmbH, Hannover, Germany; 3 Department of ABPH-REL/CCS, Established Products, Abbott Products GmbH, Hannover, Germany; 4 Department of Medicinal Chemistry/CDS, Established Products, Abbott Products GmbH, Hannover, Germany; 5 Department of Pharmacology, Cardiovascular Research Institute Maastricht, Maastricht University, Maastricht, The Netherlands

## Background

Interstitial fibrosis is the common pathophysiological mechanism that leads to end organ failure of the both the heart and the kidney. Fibrosis is characterized by an excessive accumulation of myofibroblast-derived extracellular matrix. Peritubular capillary rarefaction precedes renal fibrosis and is secondary to the loss of capillary pericytes to the interstitium. Endothelial-derived C-type natriuretic peptide (CNP) has been demonstrated to have cGMP dependent anti-fibrotic properties most likely due to the interference with pro-fibrotic TGF-β signaling and may counteract the loss of the capillary pericytes. However, natriuretic peptides like CNP are rapidly degraded by neutral endopeptidase (NEP). In a unilateral urether obstruction (UUO) mouse model for kidney fibrosis we assessed the anti-fibrotic effects of Sol-1, a new orally-active compound (Solvay) that inhibits both neutral endopeptidase and endothelin.

## Results

Mice (n=10 per group) subjected to UUO were treated for 1 week with either Solvent, SOL-1 (NEP-/ECE-inhibitor two doses), candoxatril (reference NEP inhibitor) or losartan (angiotensin AT1-receptor antagonist) and compared to sham operated animals. While the NEP inhibitors had no significant effect on body weight, food and water intake, mean blood pressure or creatinine levels, they did increase cGMP levels in the urine and affected hematopoiesis in an anti-inflammatory way. Also immunohistochemical staining revealed a marked decrease in collagen (from 1.8%±1.4% to 0.8%±0.3%, P<0.05) and α-SMA (from 8.3%±3.8% to 4.9%±1.9%, P<0.05). Moreover, α-SMA deposition in the kidney cortex was inversely correlated with cGMP elevation suggesting a NEP dependent antifibrotic effect.

To further dissect the molecular mechanisms underlying the anti-fibrotic effects of Sol-1 we performed a “Deep SAGE” transcriptome analyses total kidney samples of all treatment groups. High quality mRNA profiles were obtained with at least 8 independent samples per treatment group. The data confirms the cGMP dependent anti-fibrotic action of Sol-1 and further supports the potential therapeutic actions of this neutral endopeptidase inhibitor.

## Conclusion

A neutral endopeptidase inhibitor candoxatril and double NEP-/ECE-inhibitor SOL-1 increased cGMP levels, decreased α-SMA content in the kidney cortex and therefore showed antifibrotic properties in the mouse model of UUO.

